# Adrenal Paracoccidioidomycosis

**DOI:** 10.4269/ajtmh.20-0083

**Published:** 2020-09

**Authors:** Juan Cataño, Jessica Porras

**Affiliations:** 1 Internal Medicine Department, Infectious Diseases Section, University of Antioquia School of Medicine, Medellín, Colombia;; 2 Infectious Diseases Section, CES Clinic, Medellín, Colombia

**Figure 1.**
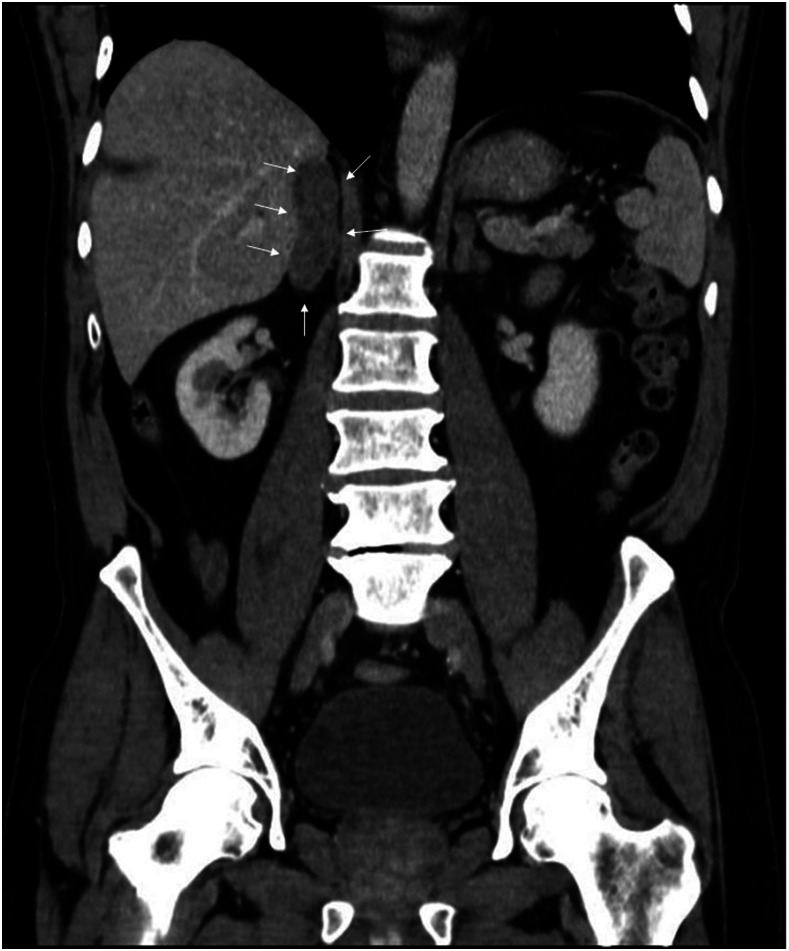
Abdominal contrasted tomography (coronal view) showing an enlarged right adrenal gland (arrows).

**Figure 2.**
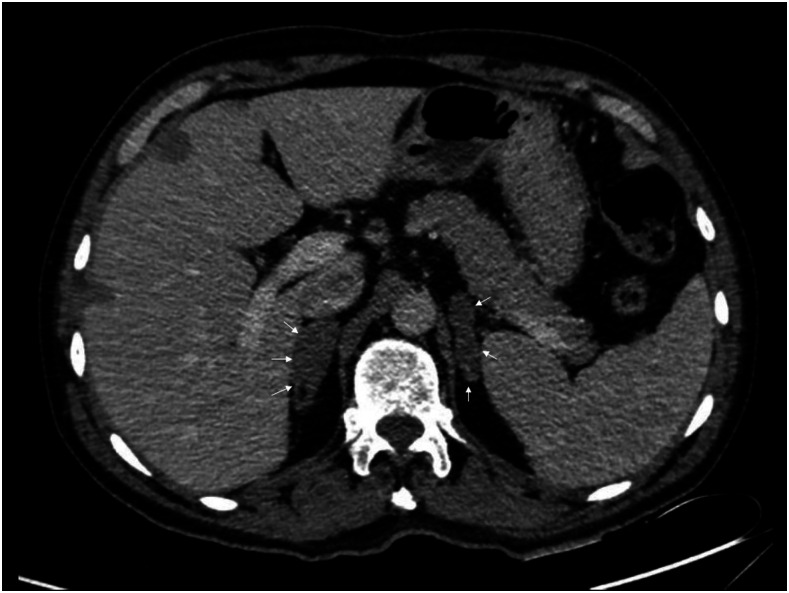
Abdominal contrasted tomography (axial view) showing a bilateral enlarged adrenal gland (arrows), predominantly the right one.

**Figure 3.**
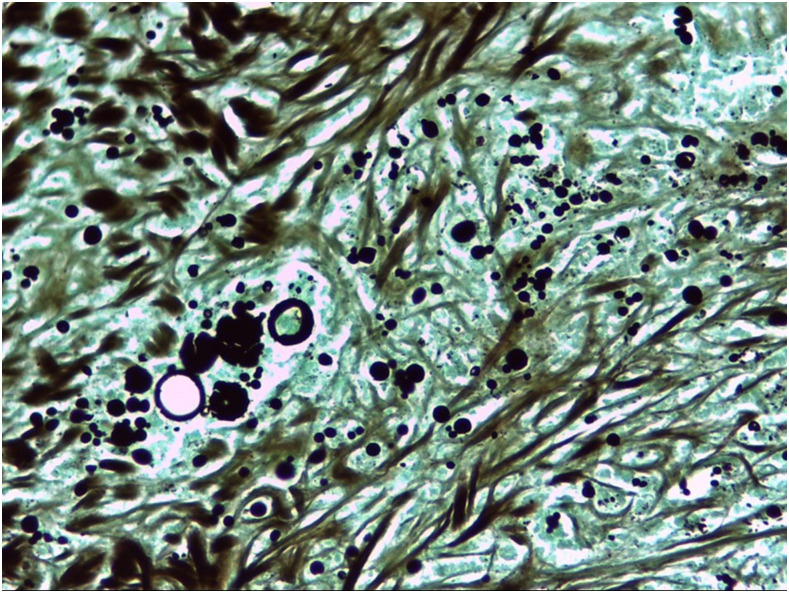
Grocott’s methenamine silver stain showing multiple, narrow-based budding yeast cells with steering wheels, consistent with *Paracoccidioides brasiliensis*. This figure appears in color at www.ajtmh.org.

A 56-year-old man with no remarkable medical history, had been working his entire life as a miner in a rural area of Colombia, consulted in the emergency department with a clinical picture of a 5-month history of asthenia, hyporexia, emesis, intermittent diarrhea, 8-kg weight loss, lethargy, and progressive darkening of the skin. On physical examination, he was found to be in regular general conditions, conscious and alert, but confused, with blood pressure of 90/60, bradycardia of 55 beats/minute, 36.0 centrigrade of temperature, and skin hyperpigmentation; the remainder of the examination did not show any other findings. Laboratory tests revealed sodium of 130 mEq/L (normal values 135–145), hyperkalemia of 5.8 mEq/dL, and acute-on-chronic kidney disease (creatinine 2.1 mg/dL). Based on symptoms, adrenal function tests were performed, showing cortisol levels of 0.97 μg/dL (normal values 5–38.4) and adrenocorticotropic hormone at 1,250 pg/mL (normal values 0–46). An abdominal contrasted tomography was performed, showing bilateral enlargement of adrenal glands, predominantly the right one (Figures 1 and 2), with the absence of calcifications. With all these findings, a right adrenal percutaneous biopsy was performed, where histopathologic Grocott’s methenamine silver stain showed multiple, narrow-based budding yeast cells with steering wheels, consistent with *Paracoccidioides brasiliensis*’s shape (Figure 3). This result was confirmed by culture, and Ziehl–Neelsen staining and mycobacterial cultures were negative. He was treated with IV hydrocortisone, oral fludrocortisone, and amphotericin B deoxycholate for one month (total = 1.5 grams), and then treated with oral itraconazole. On a follow-up visit 6 months later, he continued with hormonal replacement and itraconazole treatment, but without a crisis of adrenal insufficiency.

Paracoccidioidomycosis is a systemic fungal infection caused by the thermally dimorphic fungus *P. brasiliensis*. This disease is endemic in certain South and Central American countries, with the highest prevalence observed in Brazil.^1^ The fungus has been detected in soil; then, people who work in agriculture and live in rural areas are at a particularly high risk for infection. Human-to-human transmission has not been described, and, similar to other systemic mycoses, *P. brasiliensis* enters the host via the respiratory tract and is usually inhaled during agriculture-related activities.^2^ Depending on the patient’s immune status, it may stay inactive or spread by lymphatic and hematogenous dissemination to various secondary sites like adrenal glands.^3^
